# Supplementary material for: A Higher Healthy Eating Index Is Associated with Decreased Markers of Inflammation and Lower Odds for Being Overweight/Obese Based on a Case-Control Study
Source: Nutrients. 2022 Dec 2;14(23):5127. doi: 10.3390/nu14235127 (PMC9738448; doi:10.3390/nu14235127)
Supplement: Supplementary file 1 [file nutrients-14-05127-s001.zip › nutrients-2010344-supplementary.pdf]

Supplementary Table S1. HEI-2015 Components and Scoring Standards.

Component	Maximum points[1]	Standard for maximum score	Standard for minimum score of zero
Adequacy:			
Total Fruits[2]	5	≥0.8 cup equivalent per 1,000 kcal	No Fruit
Whole Fruits[3]	5	≥0.4 cup equivalent per 1,000 kcal	No Whole Fruit
Total Vegetables[4]	5	≥1.1 cup equivalent per 1,000 kcal	No Vegetables
Greens and Beans[4]	5	≥0.2 cup equivalent per 1,000 kcal	No Dark-Green Vegetables or Legumes
Whole Grains	10	≥1.5 cup equivalent per 1,000 kcal	No Whole Grains
Dairy[5]	10	≥1.3 cup equivalent per 1,000 kcal	No Dairy
Total Protein Foods[4]	5	≥2.5 cup equivalent per 1,000 kcal	No Protein Foods
Seafood and Plant Proteins[4,6]	5	≥0.8 cup equivalent per 1,000 kcal	No Seafood or Plant Proteins
Fatty Acids[7]	10	(PUFAs + MUFAs)/SFAs ≥2.5	(PUFAs + MUFAs)/SFAs ≤1.2
Moderation:			
Refined Grains	10	≤1.8 ounce equivalent per 1,000 kcal	≥4.3 ounce equivalent per 1,000 kcal
Sodium	10	≤1.1 grams per 1,000 kcal	≥2.0 grams per 1,000 kcal
Added Sugars	10	≤6.5% of energy	≥26% of energy
Saturated Fats	10	≤8% of energy	≥16% of energy

1 Intakes between the minimum and maximum standards are scored proportionately.
2 Includes 100% fruit juice.
3 Includes all forms except juice.
4 Includes legumes (beans and peas).
5 Includes all milk products, such as fluid milk, yogurt, cheese, and fortified soy beverages.
6 Includes seafood, nuts, seeds, soy products (other than beverages), and legumes (beans and peas).
7 Ratio of poly- and mono-unsaturated fatty acids (PUFAs and MUFAs) to saturated fatty acids (SFAs).

Supplementary Table S2. Comparison (Mean ± SD) of the macro-micronutrient intakes of the participants based on HEI, BMI, and gender groups[f].

Variables	Mean ± SD [a]											
	HEI[f]						BMI					
	Women (n=807)			Men (n=798)			Women (n=807)			Men (n=798)		
	< median (n=419)	≥ median (n=388)	p	< median (n=408)	≥ median (n=390)	p	Overweight/ Obese (n=419)	Normal weight (n=388)	p	Overweight/ Obese (n=393)	Normal weight (n=405)	p
Water (g/day)	1464 ± 566.1	1358 ± 540.6	**0.007**	1526 ± 609.7	1485 ± 561.7	0.318	1375 ± 505.3	1454 ± 604.5	**0.042**	1481 ± 562.8	1530 ± 608.8	0.242
Total energy (kcal/day)	3278 ± 515.7	2528 ± 353.3	**<0.01**	3356 ± 507.9	2497 ± 360.8	**<0.01**	2923 ± 577.3	2911 ± 587.3	0.769	2969 ± 612.5	2905 ± 619.2	0.141
Total protein (g/day)	116.0 ± 45.5	95.3 ± 32.7	**<0.01**	114.1 ± 39.8	96.6 ± 35.8	**<0.01**	106.1 ± 41.6	105.9 ± 40.8	0.941	103.8 ± 36.8	107.2 ± 40.6	0.223
Carbohydrates (g/day)	420.8 ± 112.8	303.3 ± 66.5	**<0.01**	446.5 ± 123.1	293.9 ± 63.1	**<0.01**	361.4 ± 107.8	367.4 ± 113.0	0.445	378.1 ± 120.0	365.9 ± 128.5	0.166
Total fat (g/day)	125.7 ± 45.4	103.7 ± 26.4	**<0.01**	123.8 ± 42.8	103.9 ± 32.2	**<0.01**	117.0 ± 40.2	113.1 ± 37.6	0.156	115.7 ± 41.1	112.5 ± 37.4	0.250
SFA (g/day)	49.4 ± 36.3	39.6 ± 21.8	**<0.01**	45.1 ± 30.8	40.5 ± 26.7	**0.027**	46.6 ± 33.4	42.6 ± 27.0	0.066	44.8 ± 32.6	40.9 ± 24.7	0.053
MUFA (g/day)	30.1 ± 11.7	29.5 ± 9.8	0.428	29.7 ± 11.1	29.1 ± 8.7	0.447	30.1 ± 10.8	29.4 ± 10.8	0.402	29.5 ± 9.9	29.3 ± 10.2	0.147
PUFA (g/day)	31.7 ± 18.6	26.4 ± 16.7	**<0.01**	32.9 ± 19.7	28.2 ± 19.1	**0.001**	29.3 ± 18.3	28.8 ± 17.5	0.690	29.9 ± 19.2	31.3 ± 19.9	0.328
Total fiber (g/day)	43.2 ± 17.1	43.3 ± 16.8	0.932	44.5 ± 17.8	44.6 ± 16.9	0.337	42.1 ± 17.3	44.5 ± 16.5	0.051	43.9 ± 17.3	46.4 ± 17.4	**0.040**
Soluble fiber (g/day)	0.63 ± 0.41	0.67 ± 0.42	0.295	0.62 ± 0.41	0.64 ± 0.43	0.428	0.64 ± 0.41	0.65 ± 0.40	0.782	0.60 ± 0.38	0.66 ± 0.46	**0.034**
Vitamin A (RAE/day)	675.7 ± 338.3	619.8 ± 275.3	**0.011**	720.7 ± 371.1	627.8 ± 285.7	**<0.01**	630.5 ± 291.7	668.6 ± 329.2	0.082	659.5 ± 335.0	690.5 ± 335.0	0.192
Beta-carotene (μg/day)	5255 ± 2080	5321 ± 2176	0.659	5509 ± 2234	5413 ± 2298	0.549	5222 ± 2114	5356 ± 2139	0.374	5360 ± 2255	5561 ± 2273	0.210
Alpha-carotene (μg/day)	793.7 ± 419.1	838.8 ± 408.7	0.123	782.2 ± 408.8	760.6 ± 402.8	0.453	812.5 ± 421.5	818.5 ± 407.2	0.893	787.1 ± 404.3	756.7 ± 407.1	0.291
Lutein (μg/day)	2473 ± 1349	2092 ± 948.1	**<0.01**	2498 ± 1247	2266 ± 1031	**0.004**	2302 ± 1222	2275 ± 1151	0.749	2358 ± 1135	2411 ± 1169	0.520
Lycopene (μg/day)	5149 ± 2326	4944 ± 2197	0.201	5169 ± 2259	5160 ± 2166	0.955	5034 ± 2266	5068 ± 2268	0.830	5135 ± 2222	5193 ± 2208	0.709
Vitamin C (mg/day)	152.2 ± 65.5	157.6 ± 62.7	0.232	151.1 ± 61.6	158.8 ± 57.2	0.063	155.9 ± 63.9	153.6 ± 64.6	0.606	148.3 ± 55.9	161.1 ± 62.3	**0.002**
Calcium (mg/day)	1165 ± 404.1	1198 ± 492.2	0.252	1201 ± 423.1	1214 ± 425.0	0.663	1151 ± 418.9	1212 ± 411.8	**0.035**	1183 ± 413.4	1231 ± 432.9	0.109
Iron (mg/day)	18.9 ± 5.5	19.0 ± 5.8	0.793	19.0 ± 5.7	19.0 ± 5.5	0.918	18.9 ± 5.7	19.0 ± 5.7	0.746	19.2 ± 5.7	18.8 ± 5.6	0.311
Vitamin D (IU/day)	2.01 ± 1.52	2.21 ± 1.61	0.093	2.31 ± 1.64	2.34 ± 1.55	0.776	2.09 ± 1.53	2.21 ± 1.62	0.273	2.40 ± 1.64	2.25 ± 1.55	0.200
Vitamin E (mg/day)	18.4 ± 8.4	18.4 ± 7.3	0.958	19.6 ± 8.1	19.5 ± 7.5	0.883	18.3 ± 7.6	18.4 ± 8.2	0.809	19.7 ± 8.1	19.5 ± 7.5	0.660
Thiamin (mg/day)	2.20 ± 0.87	2.18 ± 0.81	0.660	2.24 ± 0.93	2.25 ± 0.90	0.926	2.19 ± 0.86	2.19 ± 0.82	0.925	2.24 ± 0.91	2.24 ± 0.92	0.998
Riboflavin (mg/day)	2.12 ± 0.69	2.17 ± 0.79	0.293	2.21 ± 0.72	2.18 ± 0.78	0.631	2.16 ± 0.75	2.13 ± 0.73	0.590	2.18 ± 0.75	2.21 ± 0.76	0.529
Niacin (mg/day)	29.2 ± 9.7	29.9 ± 11.1	0.309	28.4 ± 9.5	30.5 ± 11.2	**0.005**	29.9 ± 10.4	29.2 ± 10.3	0.368	29.4 ± 10.2	29.5 ± 10.6	0.895
Vitamin B6 (mg/day)	2.50 ± 0.94	2.91 ± 0.87	**0.001**	2.65 ± 1.06	2.36 ± 0.89	**<0.01**	2.38 ± 0.85	2.42 ± 0.97	0.456	2.48 ± 0.96	2.53 ± 1.01	0.448
Folate (μg/day)	688.6 ± 245.0	671.2 ± 237.4	0.306	710.3 ± 215.7	693.6 ± 244.3	0.306	674.5 ± 243.9	686.5 ± 238.8	0.479	699.4 ± 235.4	704.8 ± 225.2	0.738
Vitamin B12 (μg/day)	5.62 ± 4.16	5.45 ± 2.69	0.502	5.28 ± 3.10	5.81 ± 2.92	**0.014**	5.55 ± 3.78	5.52 ± 3.24	0.894	5.55 ± 3.08	5.52 ± 2.98	0.903
Biotin (mg/day)	37.4 ± 13.5	38.6 ± 12.5	0.204	37.8 ± 13.5	38.4 ± 13.1	0.544	38.4 ± 12.8	37.6 ± 13.3	0.367	37.9 ± 13.4	38.2 ± 13.3	0.784
Pantothenic acid (mg/day)	7.52 ± 2.46	7.55 ± 2.61	0.840	7.45 ± 2.57	7.75 ± 2.71	0.101	7.44 ± 2.47	7.64 ± 2.59	0.264	7.71 ± 2.69	7.49 ± 2.60	0.233
Vitamin K (μg/day)	297.7 ± 158.5	271.1 ± 141.8	**0.012**	302.2 ± 160.9	269.8 ± 137.5	**0.002**	291.2 ± 151.2	278.0 ± 151.1	0.215	279.2 ± 146.1	293.2 ± 154.9	0.189

Magnesium (mg/day)	531.1 ± 151.3	515.6 ± 139.1	0.129	551.5 ± 167.3	518.1 ± 143.6	**0.003**	520.6 ± 145.7	526.9 ± 145.7	0.544	533.6 ± 155.9	536.7 ± 158.1	0.785
Zinc (mg/day)	15.52 ± 5.27	15.03 ± 5.21	0.179	15.29 ± 5.47	14.59 ± 4.94	0.060	15.25 ± 5.36	15.32 ± 5.12	0.863	14.72 ± 4.90	15.15 ± 5.52	0.274
Copper (µg/day)	2.64 ± 1.18	2.68 ± 1.21	0.716	2.70 ± 1.18	2.65 ± 1.26	0.510	2.68 ± 1.22	2.64 ± 1.17	0.691	2.68 ± 1.23	2.67 ± 1.22	0.838
Manganese (mg/day)	8.95 ± 3.47	8.66 ± 3.29	0.229	9.06 ± 3.04	8.66 ± 3.31	0.075	8.75 ± 3.41	8.87 ± 3.37	0.632	8.87 ± 3.14	8.86 ± 3.22	0.997
Selenium (µg/day)	127.9 ± 50.1	131.6 ± 46.8	0.279	131.0 ± 51.9	126.3 ± 47.7	0.180	129.9 ± 49.1	129.3 ± 48.0	0.862	126.4 ± 46.9	130.9 ± 52.7	0.209

[f] Categorized based on the HEI median= 48.7

[a] One-way ANOVA was used to compare nutrients intakes between groups.

BMI= Body mass index, HEI= Healthy eating index, SFA= Saturated fatty acid, MUFA= Monounsaturated fatty acid, PUFA= Polyunsaturated fatty acid.

Significant p-values are shown in **Bold**.

Supplementary Table S3. KMO and Bartlett's Test and Rotated Component Matrix of food groups based on BMI groups.

KMO and Bartlett's Test [a]

Kaiser-Meyer-Olkin Measure of Sampling Adequacy.		.461
Bartlett's Test of Sphericity	Approx. Chi-Square	271.547
	df	78
	Sig.	.000

a. Group = Obese

KMO and Bartlett's Test [a]

Kaiser-Meyer-Olkin Measure of Sampling Adequacy.		.483
Bartlett's Test of Sphericity	Approx. Chi-Square	232.726
	df	78
	Sig.	.000

a. Group = Normal

Rotated Component Matrix [a,b]

	Component	
	1	2
Grains	.545	
Legumes		.167
Starchy vegetables	-.455	-.338
Meat and proteins	.423	.135
Dairy	-.201	
Lipids	.166	
Vegetables	-.408	-.111
Fruits	.387	-.511
Nuts	-.323	.369
Sugary products		.511
Spices	.132	.591
Water	-.166	-.330
NCB	.343	

Extraction Method: Principal Component Analysis.
Rotation Method: Varimax with Kaiser Normalization.
a. Group = Obese
b. Rotation converged in 3 iterations.
NCB= Non-caloric beverages

Rotated Component Matrix [a,b]

	Component	
	1	2
Grains	.527	-.126
Legumes	.169	.292
Starchy vegetables	-.488	
Meat and proteins	.523	.166
Dairy	-.277	.117
Lipids	.138	
Vegetables	-.553	-.207
Fruits	.175	-.532
Nuts		.475
Sugary products		.535
Spices		.539
Water		
NCB	.333	-.115

Extraction Method: Principal Component Analysis.
Rotation Method: Varimax with Kaiser Normalization.
a. Group = Normal
b. Rotation converged in 3 iterations.
NCB= Non-caloric beverages

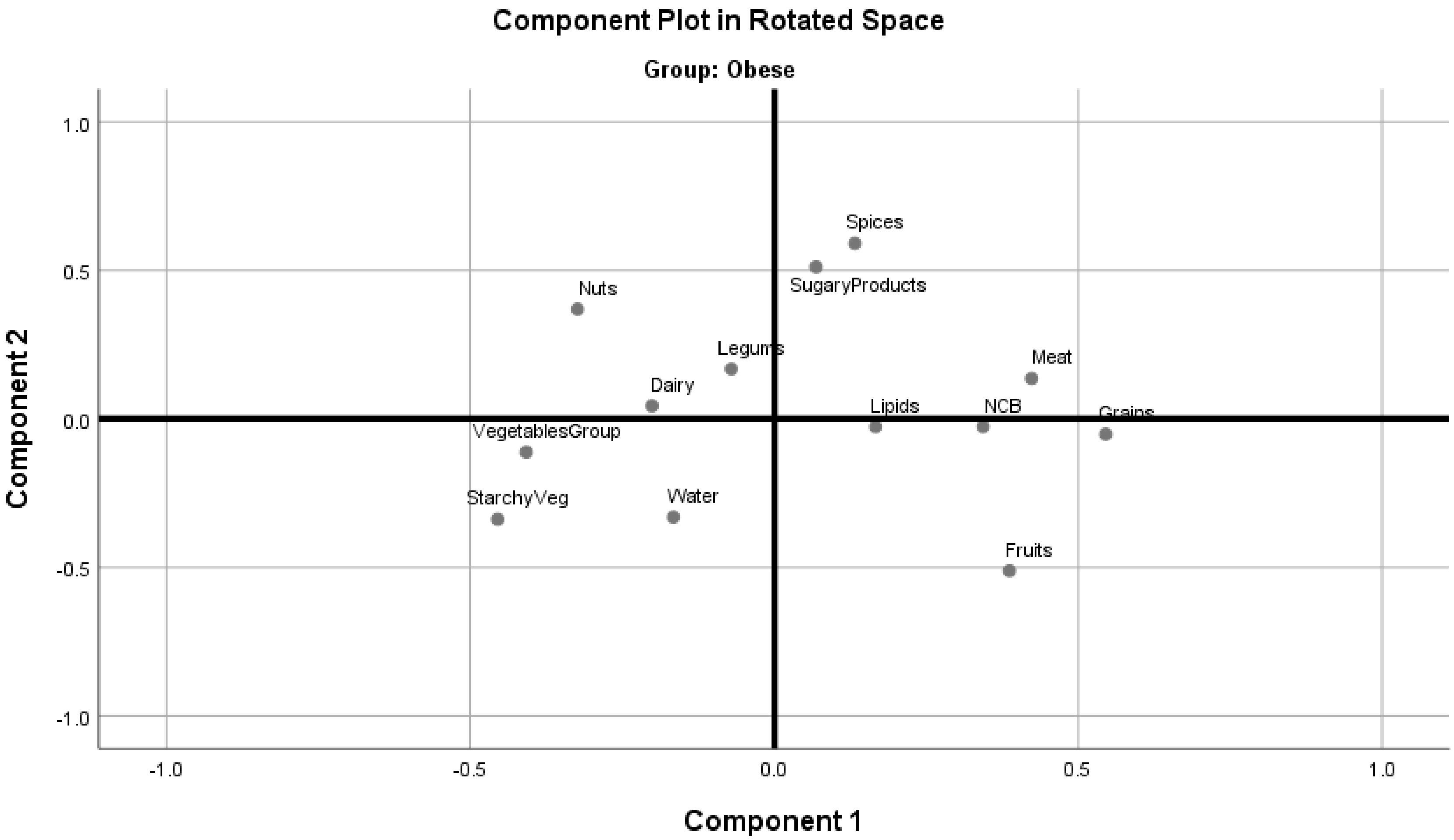

Supplementary Figure S1. Component plot in rotated space in the obese group based on BMI group.
NCB= Non-caloric beverages, BMI= body mass index

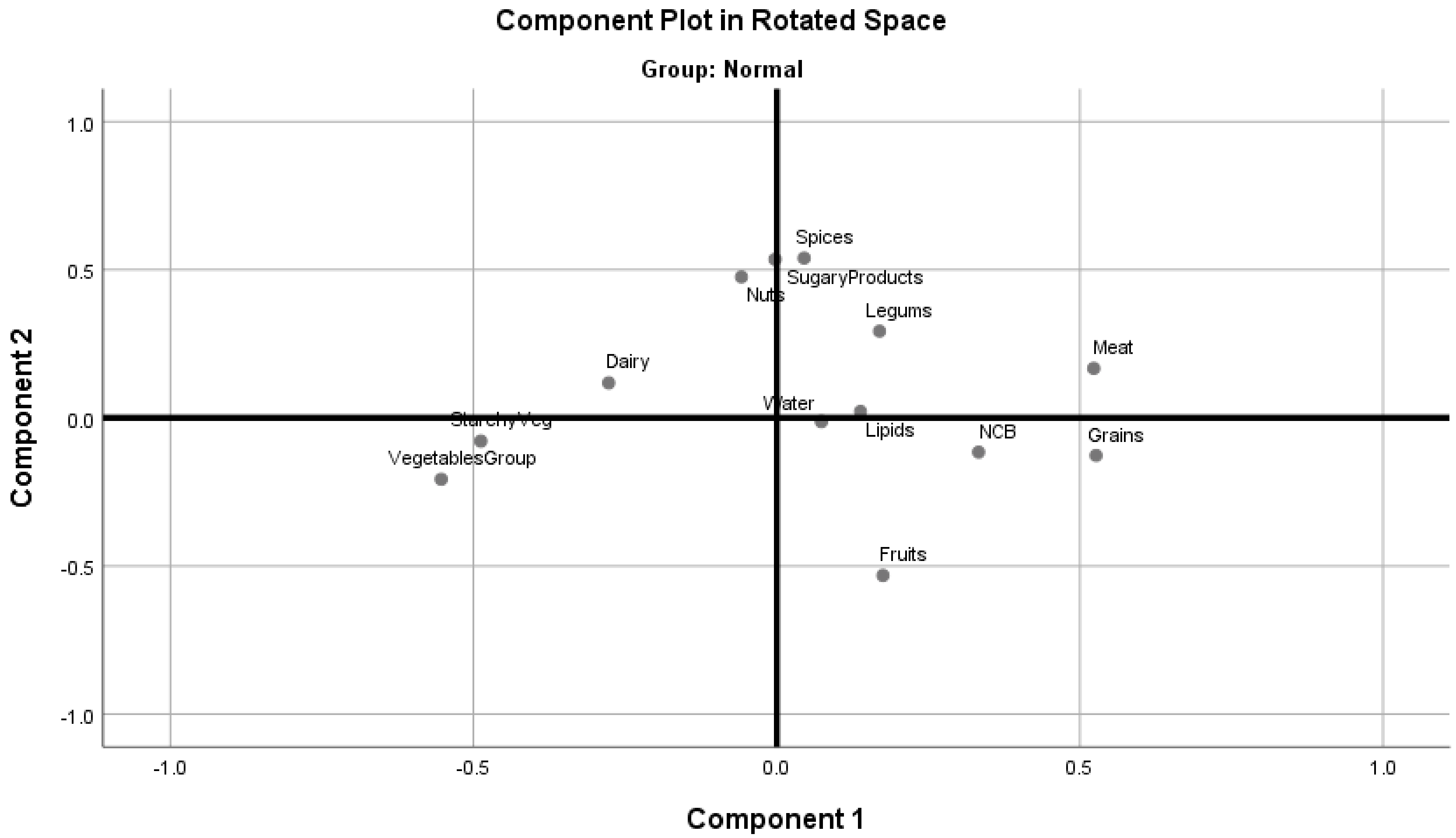

Supplementary Figure S2. Component plot in rotated space in normal weight group based on BMI group.

NCB= Non-caloric beverages, BMI= body mass index
